# 4-Isopropyl-5,5-dimethyl-2-sulfanyl-1,3,2-dioxaphosphinane 2-sulfide

**DOI:** 10.1107/S1600536812030188

**Published:** 2012-08-11

**Authors:** Sanjay K. Srivastava, Pooja Sharma, Sushil K. Gupta, Ray J. Butcher

**Affiliations:** a School of Studies in Chemistry, Jiwaji University, Gwalior 474 011, India; b Department of Chemistry, Howard University, 525 College Street NW, Washington, DC 20059, USA

## Abstract

The title compound, C_8_H_17_O_2_PS_2_, displays a distorted tetra­hedral geometry around the P atom. The P atom is part of a six-membered ring with an isopropyl group in the equatorial position. The mol­ecules are linked by S—H⋯S hydrogen bonds in the crystal packing.

## Related literature

For dithio­phospho­ric acid ligands that form metal complexes, see: Srivastava *et al.* (2010 ▶). For applications as lubricating oil additives and load-carrying capacitors, see: Jiang *et al.* (1996 ▶); Haire *et al.* (2008 ▶); Plaza *et al.* (2001 ▶). For a related structure, see: Li *et al.* (2007 ▶).
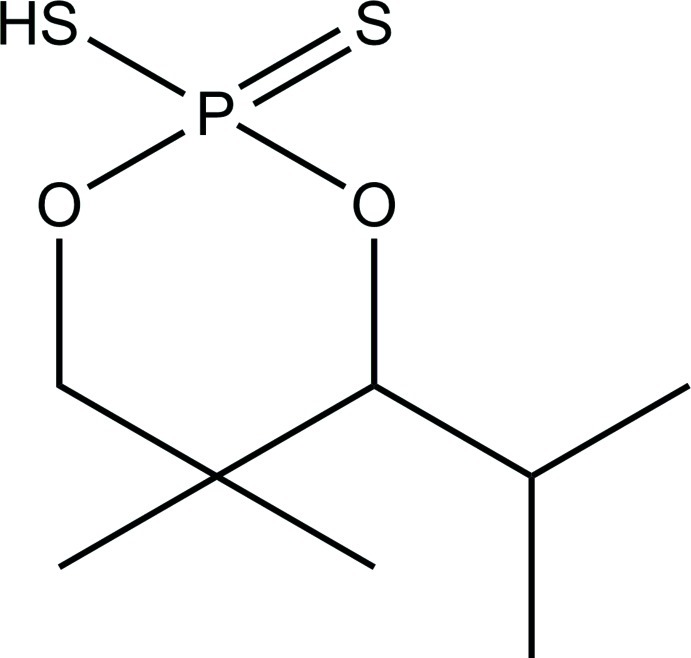


## Experimental

### 

#### Crystal data

C_8_H_17_O_2_PS_2_
*M* *_r_* = 240.31
Monoclinic, 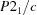

*a* = 8.2831 (2) Å
*b* = 13.1532 (4) Å
*c* = 11.5255 (3) Å
β = 104.128 (3)°
*V* = 1217.72 (6) Å^3^
*Z* = 4
Mo *K*α radiation
μ = 0.54 mm^−1^
*T* = 123 K
0.65 × 0.2 × 0.1 mm

#### Data collection

Agilent Xcalibur Ruby Gemini diffractometer
Absorption correction: multi-scan (*CrysAlis PRO*; Agilent, 2011 ▶) *T*_min_ = 0.872, *T*_max_ = 1.000
10444 measured reflections
4979 independent reflections
3999 reflections with *I* > 2σ(*I*)
*R*_int_ = 0.024

#### Refinement

*R*[*F* ^2^ > 2σ(*F* ^2^)] = 0.038
*wR*(*F* ^2^) = 0.094
*S* = 1.09
4979 reflections
123 parameters
H-atom parameters constrained
Δρ_max_ = 0.45 e Å^−3^
Δρ_min_ = −0.38 e Å^−3^

### 

Data collection: *CrysAlis PRO* (Agilent, 2011 ▶); cell refinement: *CrysAlis PRO*; data reduction: *CrysAlis PRO*; program(s) used to solve structure: *SHELXS97* (Sheldrick, 2008 ▶); program(s) used to refine structure: *SHELXL97* (Sheldrick, 2008 ▶); molecular graphics: *SHELXTL* (Sheldrick, 2008 ▶); software used to prepare material for publication: *SHELXTL*.

## Supplementary Material

Crystal structure: contains datablock(s) global, I. DOI: 10.1107/S1600536812030188/bt5962sup1.cif

Structure factors: contains datablock(s) I. DOI: 10.1107/S1600536812030188/bt5962Isup2.hkl

Supplementary material file. DOI: 10.1107/S1600536812030188/bt5962Isup3.cml

Additional supplementary materials:  crystallographic information; 3D view; checkCIF report

## Figures and Tables

**Table 1:** Hydrogen-bond geometry (Å, °)

*D*—H⋯*A*	*D*—H	H⋯*A*	*D*⋯*A*	*D*—H⋯*A*
S1—H1S⋯S2^i^	1.20	2.76	3.9456 (5)	170

Symmetry code: (i) 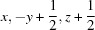.

**Fig. 1.**
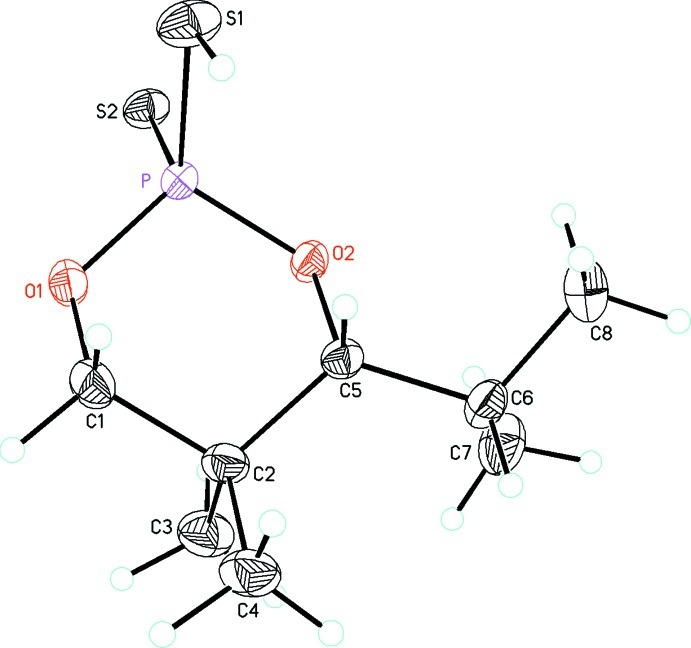
Molecular structure of the title compound showing the atom labeling scheme and 30% probability displacement ellipsoids.

**Fig. 2.**
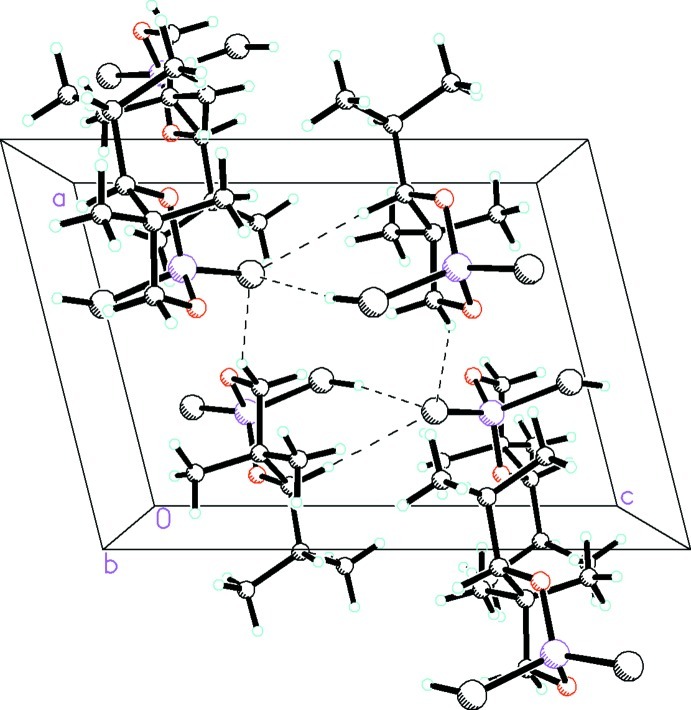
Crystal packing for (I) viewed along b axis. Dashed lines indicate an intermolecular S—H···S hydrogen bonds.

## supplementary crystallographic information

### Comment

Organo-phosphorus compounds with sulfur donors have recently drawn more attention due to their adjustable coordination ability and interesting applications as high viscosity lubricant additives(Jiang *et al.*, 1996; Haire *et al.*, 2008) and load carrying capacity (Plaza *et al.*, 2001). As part of our investigation on the organotin dithio complexes (Srivastava *et al.*, 2010), we herein report the synthesis and structure of I.

The crystal structure of I is illustrated in Fig.1. The conformation of the molecule with respect to P is distorted tetrahedral as reflected by torsion angles O2—P—O1—C1, S2—P—O2—C5 and S1—P—O1—C1 of 41.57 (10), -165.77 (7) and -74.71 (9)° respectively. The phosphorus atom is coordinated by both sulfur and oxygen atoms with the formation of a six-membered ring. The isopropyl group is in equatorial position as indicated by bond angles C5—C6—C8 [108.83 (11)°], C5—C6—C7 [115.18 (11)°] and C7—C6—C8 [110.12 (12)°]. The P—S1, P—S2 and mean P—O distances are 2.0723 (5), 1.9216 (4) and 1.5794 (9) Å, respectively, which are comparable to reported values (Li *et al.*, 2007). The molecules are stabilized by S—H···S intermolecular hydrogen bonds in the crystal packing (Table 1; Fig.2).

### Experimental

4-Isopropyl-2-mercapto-5,5-dimethyl-1,3,2-dioxaphosphinane 2-sulfide was prepared by the reaction of P_4_S_10_ (4.44 g, 0.01 mol) with *O.O*'-2,2,4-trimethyl-1,3- pentanediol(0.02 g, 0.02 mol) with stirring. The reaction was carried out in moisture free anhydrous condition and in presence of dry nitrogen. The P_4_S_10_ was slowly dissolved in glycol solution (in dry benzene) with evolution of H_2_S.The reaction mixture was warmed gently on water bath (60 - 80 °C) in order to complete the reaction. After cooling, an yellow viscous liquid was obtained which crystallizes in deep freezer overnight. White crystal suitable for X-ray analysis was obtained in 60% yield. (M.P.: 336 K). Anal. Calc. for C_8_H_17_O_2_PS_2_ (%): C,39.98; H, 7.13. Found: C 39.84; H, 7.05.

### Refinement

All H atoms were located by a Fourier map. Nevertheless, they were placed in their calculated positions and then refined using the riding model with atom—H lengths of 1.00 Å (CH),0.99 Å (CH_2_) or 0.98 Å (CH_3_). Isotropic displacement parameters for these atoms were set to 1.2 (CH or CH_2_) or 1.5 (CH_3_) times *U*_eq_ of the parent atom. The torsions angles O-P-S-H of the S-H group and C-C-C-H for the methyl groups were refined.

### Crystal data

**Table:**

C_8_H_17_O_2_PS_2_	*F*(000) = 512
*M**_r_* = 240.31	*D*_x_ = 1.311 Mg m^−^^3^
Monoclinic, *P*2_1_/*c*	Mo *K*α radiation, λ = 0.71073 Å
Hall symbol: -P 2ybc	Cell parameters from 3599 reflections
*a* = 8.2831 (2) Å	θ = 3.1–35.0°
*b* = 13.1532 (4) Å	µ = 0.54 mm^−^^1^
*c* = 11.5255 (3) Å	*T* = 123 K
β = 104.128 (3)°	Long plate, colorless
*V* = 1217.72 (6) Å^3^	0.65 × 0.2 × 0.1 mm
*Z* = 4	

### Data collection

**Table:**

Agilent Xcalibur Ruby Gemini diffractometer	4979 independent reflections
Radiation source: Enhance (Mo) X-ray Source	3999 reflections with *I* > 2σ(*I*)
Graphite monochromator	*R*_int_ = 0.024
Detector resolution: 10.5081 pixels mm^-1^	θ_max_ = 35.0°, θ_min_ = 3.1°
ω scans	*h* = −13→12
Absorption correction: multi-scan (*CrysAlis PRO*; Agilent, 2011)	*k* = −17→21
*T*_min_ = 0.872, *T*_max_ = 1.000	*l* = −18→17
10444 measured reflections	

### Refinement

**Table:**

Refinement on *F*^2^	Primary atom site location: structure-invariant direct methods
Least-squares matrix: full	Secondary atom site location: difference Fourier map
*R*[*F*^2^ > 2σ(*F*^2^)] = 0.038	Hydrogen site location: inferred from neighbouring sites
*wR*(*F*^2^) = 0.094	H-atom parameters constrained
*S* = 1.09	*w* = 1/[σ^2^(*F*_o_^2^) + (0.0372*P*)^2^ + 0.2639*P*] where *P* = (*F*_o_^2^ + 2*F*_c_^2^)/3
4979 reflections	(Δ/σ)_max_ = 0.001
123 parameters	Δρ_max_ = 0.45 e Å^−^^3^
0 restraints	Δρ_min_ = −0.38 e Å^−^^3^

### Special details

**Table:**

Geometry. All e.s.d.'s (except the e.s.d. in the dihedral angle between two l.s. planes) are estimated using the full covariance matrix. The cell e.s.d.'s are taken into account individually in the estimation of e.s.d.'s in distances, angles and torsion angles; correlations between e.s.d.'s in cell parameters are only used when they are defined by crystal symmetry. An approximate (isotropic) treatment of cell e.s.d.'s is used for estimating e.s.d.'s involving l.s. planes.
Refinement. Refinement of *F*^2^ against ALL reflections. The weighted *R*-factor *wR* and goodness of fit *S* are based on *F*^2^, conventional *R*-factors *R* are based on *F*, with *F* set to zero for negative *F*^2^. The threshold expression of *F*^2^ > σ(*F*^2^) is used only for calculating *R*-factors(gt) *etc*. and is not relevant to the choice of reflections for refinement. *R*-factors based on *F*^2^ are statistically about twice as large as those based on *F*, and *R*- factors based on ALL data will be even larger.

### Fractional atomic coordinates and isotropic or equivalent isotropic displacement parameters (Å^2^)

**Table:**

	*x*	*y*	*z*	*U*_iso_*/*U*_eq_	
P	0.80019 (4)	0.22223 (2)	0.26248 (3)	0.01693 (7)	
S1	0.89229 (5)	0.16081 (3)	0.43126 (3)	0.02970 (9)	
H1S	0.8790	0.2219	0.5057	0.036*	
S2	0.81069 (4)	0.12077 (3)	0.14460 (3)	0.02277 (8)	
O1	0.90574 (11)	0.31992 (7)	0.24915 (9)	0.02352 (19)	
O2	0.61912 (10)	0.26399 (7)	0.25326 (8)	0.01907 (17)	
C1	0.87839 (16)	0.41289 (10)	0.31086 (13)	0.0249 (3)	
H1A	0.9469	0.4682	0.2895	0.030*	
H1B	0.9153	0.4019	0.3983	0.030*	
C2	0.69484 (16)	0.44550 (10)	0.27866 (12)	0.0207 (2)	
C3	0.6409 (2)	0.47148 (12)	0.14512 (13)	0.0303 (3)	
H3A	0.7253	0.5148	0.1235	0.046*	
H3B	0.5342	0.5076	0.1284	0.046*	
H3C	0.6287	0.4087	0.0980	0.046*	
C4	0.68450 (19)	0.54015 (11)	0.35475 (13)	0.0288 (3)	
H4A	0.7635	0.5915	0.3409	0.043*	
H4B	0.7121	0.5214	0.4396	0.043*	
H4C	0.5713	0.5679	0.3322	0.043*	
C5	0.59607 (15)	0.35802 (9)	0.31797 (11)	0.0182 (2)	
H5A	0.6488	0.3453	0.4045	0.022*	
C6	0.40844 (16)	0.36803 (11)	0.30692 (12)	0.0231 (3)	
H6A	0.3888	0.4362	0.3394	0.028*	
C7	0.29883 (18)	0.36110 (15)	0.17915 (14)	0.0349 (3)	
H7A	0.1813	0.3621	0.1814	0.052*	
H7B	0.3230	0.2977	0.1421	0.052*	
H7C	0.3220	0.4191	0.1323	0.052*	
C8	0.35565 (18)	0.28763 (13)	0.38615 (14)	0.0317 (3)	
H8A	0.2379	0.2970	0.3849	0.048*	
H8B	0.4232	0.2946	0.4684	0.048*	
H8C	0.3720	0.2197	0.3559	0.048*	

### Atomic displacement parameters (Å^2^)

**Table:**

	*U*^11^	*U*^22^	*U*^33^	*U*^12^	*U*^13^	*U*^23^
P	0.01539 (13)	0.01640 (14)	0.01950 (14)	0.00176 (11)	0.00521 (11)	0.00062 (11)
S1	0.03645 (18)	0.03029 (19)	0.02098 (15)	0.00996 (15)	0.00431 (14)	0.00425 (13)
S2	0.02690 (15)	0.02011 (15)	0.02217 (15)	0.00522 (12)	0.00769 (12)	−0.00132 (12)
O1	0.0191 (4)	0.0197 (4)	0.0342 (5)	−0.0022 (3)	0.0112 (4)	−0.0017 (4)
O2	0.0157 (3)	0.0167 (4)	0.0256 (4)	0.0003 (3)	0.0065 (3)	−0.0032 (3)
C1	0.0226 (5)	0.0190 (6)	0.0339 (7)	−0.0054 (5)	0.0084 (5)	−0.0030 (5)
C2	0.0238 (5)	0.0150 (5)	0.0236 (5)	−0.0003 (4)	0.0065 (5)	−0.0002 (5)
C3	0.0390 (8)	0.0260 (7)	0.0273 (6)	0.0021 (6)	0.0106 (6)	0.0053 (6)
C4	0.0352 (7)	0.0171 (6)	0.0344 (7)	−0.0012 (5)	0.0092 (6)	−0.0038 (5)
C5	0.0186 (5)	0.0160 (5)	0.0204 (5)	0.0010 (4)	0.0055 (4)	−0.0015 (4)
C6	0.0186 (5)	0.0234 (6)	0.0282 (6)	0.0027 (5)	0.0074 (5)	−0.0043 (5)
C7	0.0197 (6)	0.0500 (10)	0.0332 (7)	0.0035 (6)	0.0027 (5)	0.0027 (7)
C8	0.0263 (6)	0.0393 (8)	0.0332 (7)	−0.0052 (6)	0.0141 (6)	−0.0030 (6)

### Geometric parameters (Å, º)

**Table:**

P—O2	1.5762 (9)	C3—H3C	0.9800
P—O1	1.5826 (10)	C4—H4A	0.9800
P—S2	1.9216 (5)	C4—H4B	0.9800
P—S1	2.0723 (5)	C4—H4C	0.9800
S1—H1S	1.2000	C5—C6	1.5337 (17)
O1—C1	1.4599 (17)	C5—H5A	1.0000
O2—C5	1.4804 (15)	C6—C8	1.529 (2)
C1—C2	1.5355 (18)	C6—C7	1.533 (2)
C1—H1A	0.9900	C6—H6A	1.0000
C1—H1B	0.9900	C7—H7A	0.9800
C2—C3	1.5328 (19)	C7—H7B	0.9800
C2—C4	1.5372 (18)	C7—H7C	0.9800
C2—C5	1.5426 (18)	C8—H8A	0.9800
C3—H3A	0.9800	C8—H8B	0.9800
C3—H3B	0.9800	C8—H8C	0.9800
			
O2—P—O1	104.46 (5)	H4A—C4—H4B	109.5
O2—P—S2	113.64 (4)	C2—C4—H4C	109.5
O1—P—S2	111.95 (4)	H4A—C4—H4C	109.5
O2—P—S1	108.99 (4)	H4B—C4—H4C	109.5
O1—P—S1	108.79 (4)	O2—C5—C6	106.41 (10)
S2—P—S1	108.85 (2)	O2—C5—C2	109.41 (10)
P—S1—H1S	109.5	C6—C5—C2	120.72 (11)
C1—O1—P	118.53 (8)	O2—C5—H5A	106.5
C5—O2—P	119.65 (7)	C6—C5—H5A	106.5
O1—C1—C2	112.24 (10)	C2—C5—H5A	106.5
O1—C1—H1A	109.2	C8—C6—C7	110.12 (12)
C2—C1—H1A	109.2	C8—C6—C5	108.83 (11)
O1—C1—H1B	109.2	C7—C6—C5	115.18 (11)
C2—C1—H1B	109.2	C8—C6—H6A	107.5
H1A—C1—H1B	107.9	C7—C6—H6A	107.5
C3—C2—C1	109.50 (11)	C5—C6—H6A	107.5
C3—C2—C4	110.43 (11)	C6—C7—H7A	109.5
C1—C2—C4	106.15 (11)	C6—C7—H7B	109.5
C3—C2—C5	114.56 (11)	H7A—C7—H7B	109.5
C1—C2—C5	106.59 (10)	C6—C7—H7C	109.5
C4—C2—C5	109.22 (11)	H7A—C7—H7C	109.5
C2—C3—H3A	109.5	H7B—C7—H7C	109.5
C2—C3—H3B	109.5	C6—C8—H8A	109.5
H3A—C3—H3B	109.5	C6—C8—H8B	109.5
C2—C3—H3C	109.5	H8A—C8—H8B	109.5
H3A—C3—H3C	109.5	C6—C8—H8C	109.5
H3B—C3—H3C	109.5	H8A—C8—H8C	109.5
C2—C4—H4A	109.5	H8B—C8—H8C	109.5
C2—C4—H4B	109.5		
			
O2—P—O1—C1	41.56 (10)	P—O2—C5—C2	56.81 (12)
S2—P—O1—C1	164.95 (8)	C3—C2—C5—O2	61.00 (14)
S1—P—O1—C1	−74.71 (9)	C1—C2—C5—O2	−60.27 (13)
O1—P—O2—C5	−43.49 (10)	C4—C2—C5—O2	−174.55 (10)
S2—P—O2—C5	−165.77 (7)	C3—C2—C5—C6	−62.90 (16)
S1—P—O2—C5	72.65 (9)	C1—C2—C5—C6	175.83 (11)
P—O1—C1—C2	−54.83 (14)	C4—C2—C5—C6	61.55 (15)
O1—C1—C2—C3	−63.68 (14)	O2—C5—C6—C8	72.23 (13)
O1—C1—C2—C4	177.11 (11)	C2—C5—C6—C8	−162.46 (12)
O1—C1—C2—C5	60.77 (14)	O2—C5—C6—C7	−51.96 (15)
P—O2—C5—C6	−171.25 (8)	C2—C5—C6—C7	73.35 (16)

### Hydrogen-bond geometry (Å, º)

**Table:**

*D*—H···*A*	*D*—H	H···*A*	*D*···*A*	*D*—H···*A*
S1—H1*S*···S2^i^	1.20	2.76	3.9456 (5)	170

Symmetry code: (i) *x*, −*y*+1/2, *z*+1/2.
